# Improving Fab’ fragment retention in an autonucleolytic *Escherichia coli* strain by swapping periplasmic nuclease translocation signal from OmpA to DsbA

**DOI:** 10.1007/s10529-017-2425-z

**Published:** 2017-09-05

**Authors:** Desmond M. Schofield, Ernestas Sirka, Eli Keshavarz-Moore, John M. Ward, Darren N. Nesbeth

**Affiliations:** 1 Department of Biochemical Engineering, University College London, Bernard Katz Building, London, WC1E 6BT UK; 2 Centre for Translational Omics, UCL Institute of Child Health & Great Ormond Street Hospital, 30 Guilford Street, London, WC1N 1EH UK

**Keywords:** Autonucleolytic, Bioprocess, Fab’ fragment, Leakage, Nuclease, Periplasm

## Abstract

**Objectives:**

To reduce unwanted Fab’ leakage from an autonucleolytic *Escherichia coli* strain, which co-expresses OmpA-signalled Staphylococcal nuclease and Fab’ fragment in the periplasm, by substituting in Serratial nuclease and the DsbA periplasm translocation signal as alternatives.

**Results:**

We attempted to genetically fuse a nuclease from *Serratia marcescens* to the OmpA signal peptide but plasmid construction failed, possibly due to toxicity of the resultant nuclease. Combining Serratial nuclease to the DsbA signal peptide was successful. The strain co-expressing this nuclease and periplasmic Fab’ grew in complex media and exhibited nuclease activity detectable by DNAse agar plate but its growth in defined medium was retarded. Fab’ coexpression with Staphylococcal nuclease fused to the DsbA signal peptide resulted in cells exhibiting nuclease activity and growth in defined medium. In cultivation to high cell density in a 5 l bioreactor, DsbA-fused Staphylococcal nuclease co-expression coincided with reduced Fab’ leakage relative to the original autonucleolytic Fab’ strain with OmpA-fused staphylococcal nuclease.

**Conclusions:**

We successfully rescued Fab’ leakage back to acceptable levels and established a basis for future investigation of the linkage between periplasmic nuclease expression and leakage of co-expressed periplasmic Fab’ fragment to the surrounding growth media.

## Introduction

**Fig. 1:**
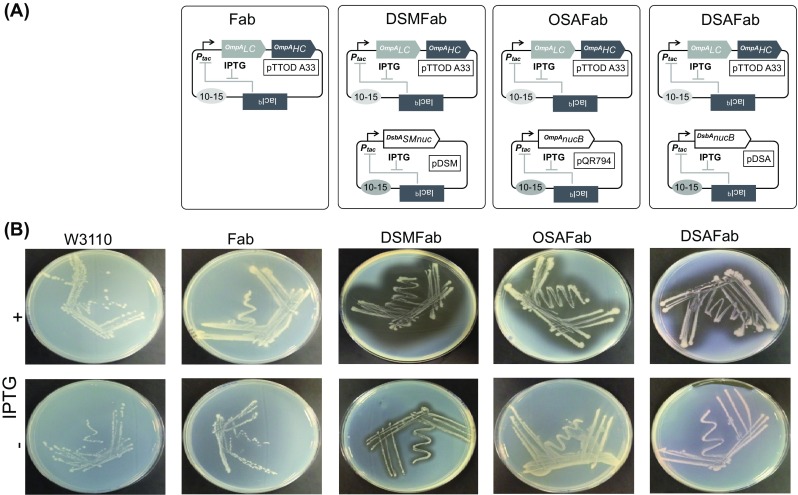
Strains in this study and their nuclease activity. **a** From left, the Fab strain harbours only the plasmid pTTOD-A33 with p15A origin of replication (ori) directing a copy number of 10–15 replicons per cell (*grey oval*). A copy of the lacIq gene encodes expression of lacI that represses the *P*_*tac*_ promoter unless IPTG is present. ORFs encoding Fab’ light and heavy chains fused to the OmpA periplasm translocation signal, ^*OmpA*^ *LC* and ^*OmpA*^ *HC* are under the control of a single *P*_*tac*_ promoter within a dicistronic expression cassette. The DSMFab strain harbours both the plasmids pTTOD-A33 and pDSM, which has a compatible RSF1010 *ori* that directs replication of 10–15 copies per cell (*grey oval*). An ORF encoding *S. marcescens* nuclease nuclease fused to the DsbA signal (^DsbA^SMnuc) is under control of the *P*_*tac*_ promoter. A copy of the lacIq gene in pDSM also coordinates IPTG-inducible expression of ^DsbA^SMnuc from the *P*_*tac*_ promoter. The OSAFab strain harbours pTTOD-A33 and pQR794, which encodes the *S. aures* nucB nuclease fused to the OmpA signal (^OmpA^nucB) under control of the *P*_*tac*_ promoter. The DSAFab strain harbours pTTOD-A33 and pDSA, which encodes the *S. aureus* nucB nuclease fused to the DsbA signal (^DsbA^nucB) under control of the *P*_*tac*_ promoter. **b** Unmodified W3110 cells and the Fab, DSMFab, OSAFab and DSAFab strains were streaked onto DNAse agar plates with (+) and without (−) IPTG present. After overnight incubation plates were flooded with 2 M HCl and photos taken of the resultant cloudy pattern of DNA precipitation. *Clear halos* indicate nuclease activity

Approaching half of all global blockbuster drugs are currently antibody-based recombinant proteins, a growing number of which are Fab’ antibody fragments. When expressed in *Escherichia coli*, genetically-appended peptide signals are commonly used to direct translocation of Fab’ fragment heavy and light chains to the oxidising environment of the periplasmic space where they can form disulphide bonded heterodimers (Ukkonen et al. 2013). Balasundaram et al. (2009) engineered *E. coli* so that a commercial anticancer Fab’ fragment (UCB Celltech UK, Slough, UK) was co-expressed with recombinant Staphylococcal nuclease (Fig. 1) to generate an autonucleolytic Fab’ production strain, termed ‘Fab’ Nuc’ previously and ‘OSAFab’ in this study.

In the OSAFab strain, both Fab’ and nuclease were genetically fused to an OmpA periplasmic translocation peptide sequence and their transcription was controlled by *P*_*tac*_ promoters (de Boer et al. 1983). Cytoplasmic nuclease expression is, however, lethal in *E. coli* (Ahrenholtz et al. 1994) but in the OSAFab strain periplasmic localisation safely sequesters the nuclease from the host genome, enabling normal cell growth. The *E. coli* OmpA periplasm translocation signal directs transport to the periplasmic space via the general secretion (SEC) route (Pugsley 1993). Native *E. coli* proteins translocated by the SEC-route are understood to be translated in the cytosol where they reside briefly before post-translational translocation to the periplasmic space via the SEC pore complex (Pugsley 1993). Upon homogenisation of the OSAFab *E. coli* strain the Staphylococcal nuclease gained access to and degraded host DNA, decreasing the viscosity of the bioprocess stream and improving its clarification performance.

One unwanted property of the Balasundaram et al. (2009) OSAFab strain, relative to the parental Fab strain without nuclease, was an increased propensity for leakage of Fab’ fragment to the growth medium during cultivation to high cell density (reported by Nesbeth et al. 2012). This observation suggested the conclusion that, directly or indirectly, the co-expression of nuclease resulted in the increased level of Fab’ leakage, by an as yet unknown mechanism. The intent of the study reported here was to test the hypothesis that, as well as nuclease presence, also the type of nuclease and the type of periplasm translocation signal peptide may also be factors that impact Fab’ leakage.

We chose the DsbA signal as an alternative to OmpA. The *E. coli* DsbA signal (Luirink and Dobberstein 1994) directs translocation via the signal recognition particle (SRP) route in which translation of nascent polypeptides is paused by SRP binding, to form an SRP-ribosome nascent chain (RNC) complex, followed by migration to the FtsY receptor element of the SecYEG pore where translation re-starts concurrently with translocation to the periplasmic space (Avdeeva et al. 2002; Park et al. 2014; Yosef et al. 2010). This contrasts with translocation via OmpA, in which nascent polypeptides are predicted to reside in the cytoplasm prior to translocation (Movva et al. 1980). We reasoned that the more divergent our choice of alternative signal was, in terms of translocation mechanism, the greater the likelihood of observing an impact on strain phenotype.

As an alternative to Staphylococcal nuclease we chose the nuclease of *Serratia marcescens* which is widely utilised commercially as Benzonase (Ball et al. 1987). We had previously compared the effectiveness of exogenously added Benzonase versus cellularly expressed Staphylococcal nuclease for clearing DNA from process streams (Balasundaram et al. 2009). As such we were interested to take the next logical step and test whether cellularly expressed Benzonase impacted strain performance and resulted in nuclease activity.

To test the hypothesis that nuclease and signal peptide choice can impact Fab’ leakage, we attempted to make, and measure Fab’ leakage in, three new strains (two of which are illustrated in Fig. 1). Attempts were made to construct *E. coli* strains in which the following nucleases would be co-expressed with a periplasmic Fab’ fragment: Staphylococcal nuclease fused to the DsbA signal in the strain, ‘DSAFab’, Serratial nuclease fused to the OmpA signal in ‘OSMFab’ and Serratial nuclease fused to the DsbA signal in ‘DSMFab’.

## Materials and methods

All chemicals were purchased from Sigma unless stated otherwise, and were of analytical grade.

### Plasmid construction

Figure 1 sets out the relevant genes present in each plasmid. DNA encoding *Staphylococcus aureus* nuclease fused to the DsbA secretion signal was provided by Eurogentec (Liege, Belgium) in a pUC57 vector then subcloned into pMMB67EH (Furste et al. 1986) between *Eco*RI and *Pst*I restriction sites. DNA encoding *S. marcescens* nuclease was amplified from locus M19495 (GenBank) in genomic DNA (Bergkessel and Guthrie 2013) using a forward primer designed to anneal downstream of the predicted *S. marcescens* secretion signal and introduce an in-frame *Hin*dIII site (underlined), CGAAGCTTGGACACGCTCGAATCCATCGACAACTGCGCGG, and a reverse primer designed to introduce an *Eco*RI site (underlined) downstream of the stop codon, CGAATTCAGTTTTTGCAGCCCATCAACTCCGGCAGAACGCCCGG. Attempts were made to subclone this fragment into a plasmid encoding an OmpA secretion signal with an in-frame *Hin*dIII site positioned at the final codon of the signal. The *S. marcescens* nuclease fragment was successfully ligated downstream of a DNA fragment encoding the DsbA signal with an in-frame *Hin*dIII site positioned at the final codon sequence before being subcloned into pMMB67EH. pTTOD A33 encoding Fab’ fragment was donated by UCB Celltech and pQR794, encoding *S. aureus* nuclease fused to the OmpA secretion signal, was constructed as previously described (Nesbeth et al. 2012). Bacterial cell transformations were performed using standard molecular biology techniques.

### Plate assay of nuclease activity

DNase agar plates were flooded with 2 M HCl to form a cloudy DNA precipitate after overnight growth. Nuclease activity was evidenced by precipitate-free zones of clearing around colonies (Cooke et al. 2003).

### Shake-flask cultivation

20 μl of a working cell bank glycerol stock was used to inoculate 200 ml lysogeny broth in a 1 l shake flask. It was grown at 37 °C with shaking at 250 rpm. For defined media cultivation, 40 ml of this culture was taken when OD_600_ reached 1, and used to inoculate 360 ml modified defined media. This modified defined medium (Li et al. 2012) contained 5.2 g (NH_4_)_2_SO_4_ l^−1^, 4.4 g NaH_2_PO_4_·H_2_O l^−1^, 3.4 g Na_2_HPO_4_ l^−1^, 4.03 g KCl l^−1^, 1.04 g MgSO_4_·7H_2_O l^−1^, 4.16 g citric acid monohydrate l^−1^, 0.25 g CaCl_2_·2H_2_O l^−1^, 112 g anhydrous glycerol l^−1^ and 1% (v/v) trace elements solution. The trace elements solution consisted of 104 g l^−1^ citric acid monohydrate, 5.22 g l^−1^ CaCl_2_·2H_2_O, 2.06 g l^−1^ ZnSO_4_·7H_2_O, 2.72 g l^−1^ MnSO_4_.4H_2_O, 0.81 g l^−1^ CuSO_4_·5H_2_O, 0.42 g l^−1^ CoSO_4_·7H_2_O, 10.06 g l^−1^ FeCl_3_·6H_2_O, 0.03 g l^−1^ H_3_BO_3_, and 0.02 g l^−1^ Na_2_MoO_4_·2H_2_O. The medium pH was adjusted to 6.95 with 15% (w/v) NH_4_OH. This defined media culture was then grown at 30 °C, 200 RPM orbital shaking.

### Bioreactor cultivation

400 ml defined media culture from the shake cultivation described above was inoculated into 4.5 l of the same modified defined media as above in a New Brunswick BioFlo 110 7.5 l bioreactor. 40:60 O_2_/N_2_ gas blending was used when necessary to maintain dissolved O_2_ at 30%. 15% (w/v) NH_4_OH and 20% (v/v) H_2_SO_4_ were used to maintain pH at 6.95. The culture was maintained at 30 °C for ~32 h. After approx. 36 h post-inoculation the culture temperature was reduced to 25 °C, glycerol fed to the fermenter as described by Perez-Pardo et al. (2011) and Fab’ production induced by addition of IPTG. Samples were stored overnight (16–20 h) at −20 ^°^C before further experimentation. For dry cell weight determination, supernatant was removed and pellet dried in an oven until constant weight measured, typically after 24 h drying time.

### Fab’ fragment extraction and assay

Total Fab’ was extracted from culture samples by transferring 1.5 ml samples to capped borosilicate glass tubes for complete cell disruption by adaptive focused acoustics (AFA) as described by Nesbeth et al. (2012). Briefly, glass sample tubes were submerged in an 8 °C (±4 °C) degassed water bath and subjected to acoustic radiation of 85 W intensity by an acoustic energy ‘burst’ consisting of 500 active energy cycles and 2000 ‘off’ cycles. The disrupted sample was centrifuged for 20 min at ~10,000×*g* at 15 °C to remove cell debris. Supernatant was filtered through a 0.22 µM syringe and transferred to 2 ml crimp-top vials fitted with 0.1 ml inserts (VWR International Limited, Leicester, UK). Growth medium samples were prepared by centrifuging 1.5 ml broth samples for 10 min (omitting disruption steps) and retaining the supernatant to be processed as described above.

### Quantitation of Fab’ levels

Fab’ was quantified using a 1 ml capacity protein G Hi-Trap column (G. E. Healthcare) in a HPLC as described by Nesbeth et al. (2012). Purified Fab’ standards of known concentration were generated in the same manner as previous studies by García-Arrazola et al. (2005) in which ELISA was used for quantitation. Fab’ was immobilised from samples with packed bed Protein A affinity chromatography using an AKTAprime system (Amersham Biosciences UK Ltd., UK) and an XK50 column (50 mm × 70 mm, 50 ml column volume) packed with Protein A Sepharose 4 Fast Flow matrix (both from GE Healthcare). An operating flow rate of 35 ml min^−1^ was used throughout. Glycine/glycinate was added to the column to 1 M (pH 7.5). The column was then equilibrated with 15 column volumes of equilibration buffer (1 M glycine, pH 8). Fab’ elution was performed using a method described previously by Bowering et al. (2002). Briefly, Fab’ fractions were pooled and buffer-exchanged into storage buffer (100 mM sodium acetate, 125 mM NaCl, 0.02% w/v sodium azide, pH 5.5) using a stirred ultrafiltration cell 8400 (Amicon) with a 20 kDa cut-off Ultracel YM regenerated cellulose membrane (Millipore). Purified Fab’ levels were calibrated using previous purified batches as reference. Historically, previous batches of purified Fab’ have ultimately been calibrated against batches measured by ELISA. The research-validated (Felinger and Guiochon 2001) software package ‘Chemstation’ (Agilent technical manual G2070-91126) was used to measure the 220 nm peak area to quantify Fab’.

## Results and discussion

### Serratial nuclease expression in *E. coli* impacts cell viability

We attempted construction of a plasmid encoding a Serratial nuclease fused to the OmpA signal controlled by the *P*_*tac*_ promoter. None of our approaches were able to yield cells harbouring the desired construct. A control plasmid encoding the Serratial nuclease open reading frame with no promoter upstream was readily constructed and propagated in *E. coli*. This suggests the Serratial nuclease coding sequence itself is not toxic to *E. coli* and that it is the expressed nuclease that is responsible for cytotoxicity. Serratial nuclease expression in *E. coli* is non-lethal (Ball et al. 1987; Biedermann et al. 1989, 1990; Friedhoff et al. 1994) if the native secretion signal was present and lethal (Ahrenholtz et al. 1994; Li and Wu 2009) or if there was no secretion signal at all. Consideration of the previous reports, and our own observation that Serratial nuclease fused to OmpA was lethal to cells, led us to conclude that the OmpA signal was non-functional when fused to Serratial nuclease and that this resulted in toxicity.

### Serratial nuclease expression in the *E. coli* periplasm impacts cell growth

Serratial nuclease was fused to the DsbA signal (Schierle et al. 2003) in the plasmid, pDSM, as was Staphylococcal nuclease in the plasmid, pDSA (Fig. 1a). The previously constructed plasmid, pQR794 (Nesbeth et al. 2012), encoded Staphylococcal nuclease fused to the OmpA signal. The nuclease expression plasmids and pTTOD-A33 were used to successfully generate four strains; the Fab strain encoding Fab’ fragment only, the OSAFab strain encoding Fab’ and OmpA-signalled Staphylococcal nuclease, the DSAFab strain encoding Fab’ and DsbA-signalled Staphylococcal nuclease and the DSMFab strain encoding Fab’ fragment and DsbA-signalled Serratial nuclease (Fig. 1a).

We tested cells for periplasmic nuclease activity using DNAse agar plates. As expected, nuclease activity was absent from the plasmid-free parental W3110 strain and the Fab strain (Fig. 1b). Periplasmic routing of nuclease via both the OmpA signal (OSAFab strain) and DsbA signal (DSAFab and DSMFab strains) resulted in nuclease activity. This indicated that translocation of active nuclease to the periplasmic space was achieved by both routes.

Unexpectedly, DNAse agar plate assay data (Fig. 1b) also showed a halo of nuclease activity for strain DSMFab even in the absence of IPTG induction. Suspecting leakiness in the *P*_*tac*_ promoter (Amann et al. 1983), we re-sequenced the *P*_*tac*_ region of the pDSM plasmid and found the promoter sequence to be unchanged. As such no greater degree of leakiness is expected from the DSMFab strain than for the other two nuclease-expressing strains, OSAFab and DSAFab. A possible explanation for this unexpected nuclease activity is that the activity of the Serratial nuclease was significantly higher than that of either Staphylococcal nuclease variant. As such, even if all three nucleases are expressed to the same, basal degree in the absence of IPTG, the small quantity of the Serratial nuclease could still be sufficient to effect observable DNA hydrolysis (Fig. 1b).

**Fig. 2:**
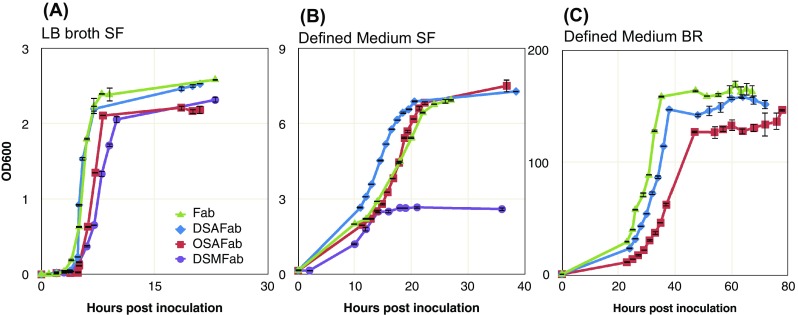
Growth performance of strains used in this study. Fab’, DSMFab, OSAFab and DSAFab strains were used to inoculate: **a** 200 ml of LB broth in 1 l shake flasks (SF) and **b** 400 ml defined media in 2 l shake flasks. **c** Failure of DSMFab growth to sufficient cell density meant that only Fab, OSAFab and DSAFab strains were used to inoculate 4 l defined media in a New Brunswick BioFlo 110 7.5 l bioreactor (BR). Optical density measurements were taken at the indicated time points. Key in graph *A* applies to graphs *B* and *C* also. *Error bars* show standard deviation across two biological repeats

All strains grew well in shake-flasks using complex media (Fig. 2a) but defined media revealed growth retardation of the DSMFab strain (Fig. 2b). Previous reports (Ball et al. 1987; Biedermann et al. 1989, 1990; Friedhoff et al. 1994) in which wildtype Serratial nuclease expression was non-lethal to *E. coli* exclusively used complex media for cell cultivation. Dragosits et al. (2012) indicated that, relative to complex media, growth in defined media increase cell stress in *E. coli*. This growth retardation in the DSMFab strain suggests combining Serratial nuclease expression with the stress burden of growth in chemically defined media was deleterious to cell growth.

It is commonplace in industrial settings to discontinue cell cultivation procedures if cell growth performance is sub-optimal during the ‘seed train’ of shake flask cultivation prior to inoculation of bioreactor cultures. In order to focus on industrially-relevant cultivation environments the DSMFab strain was not taken forward for bioreactor cultivation due to the growth retardation it showed relative to the other strains grown in defined media in shake flasks (Fig. 2b). We continued experimentation with only the Fab, OSAFab and DSAFab strains. These strains were grown to high cell density in a New Brunswick BioFlo 110 7.5 l bioreactor with variance between strains falling within the 20% level typical of bioreactor cultivation repeats (Fig. 2c).

### Fab’ retention improved by swapping Staphylococcal nuclease translocation signal

**Fig. 3:**
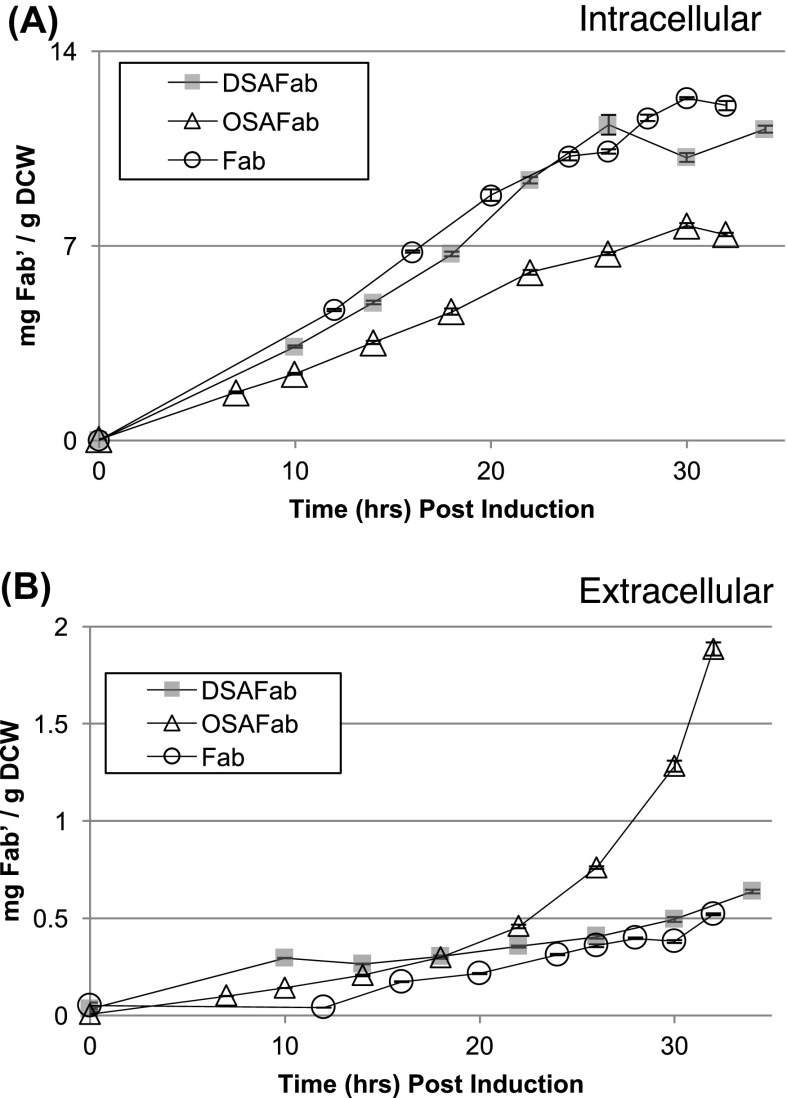
Fab’ fragment production and leakage. **a** Specific, post-induction, intracellular Fab’ levels measured for the Fab, OSAFab and DSAFab strains during bioreactor cultivation. **b** Specific, post-induction, Fab’ levels measured in the growth medium for Fab, OSAFab and DSAFab strains during bioreactor cultivation

Specific yield of intracellular Fab’ was lower for the OSAFab strain than the Fab strain, with a difference that widened from 20 h post-induction onward (Fig. 3a). In contrast, intracellular Fab’ production in the DSAFab strain matched well the Fab’ levels achieved by the nuclease-free Fab strain (Fig. 3a). All three strains showed Fab’ leakage over the first 20 h post-induction (Fig. 3b) but at approx. 22 h post-induction the OSAFab strain showed a steep increase in Fab’ leakage to the surrounding growth media. The DSAFab strain showed a Fab’ leakage profile similar to the Fab strain, in which no steep increase occurred. Future investigations will be required to ascertain the biomolecular events causing the increased leakage of Fab’ from the OSAFab strain compared to the DSAFab and Fab strains.

## Conclusions

Having previously observed that nuclease co-expression coincides with increased Fab’ leakage from an *E. coli* production strain (Nesbeth et al. 2012) we sought here to test the hypothesis that the type of nuclease and signal peptide chosen for nuclease co-expression could also influence Fab’ leakage. We considered that testing this hypothesis would be an important first step toward future work to dissect the underlying mechanisms responsible for the leakage.

Construction of a plasmid encoding Serratial nuclease with an OmpA signal failed, most likely due to the toxicity of this protein. Secreted bacterial nucleases can provide a competitive advantage to certain bacterial cells by being toxic to other bacteria (Cao et al. 2016). If the OmpA signal is non-functional when attached to Serratial nuclease, this would mean the nuclease is in effect in a mature state within the host *E. coli* cytoplasm and therefore likely to be cytotoxic by having access to genomic DNA.

Exchanging the OmpA signal to DsbA for the Serratial nuclease resulted in a viable strain, however the strain only grew slowly in chemically defined media. We conclude this slow growth is due to the combination of (i) the increased metabolic burden exerted by defined media relative to complex media and (ii) burdensome effects of a higher level of activity in the ^DsbA^SMnuc nuclease, compared to ^OmpA^nucB and ^DsbA^nucB, which led to a greater requirement for efficient periplasmic translocation for cell maintenance. Addressing these two pressures retarded cell growth in strain DSMFab, whereas DSAFab and OSAFab strains face only the former pressure of adaptation to defined media so achieved higher growth rates.

For the nuclease strains in this study, DSMFab, DSAFab and OSAFab, the cellular machineries involved in translocation of proteins from the cytosol to the periplasm are likely to be crucial to cell survival, due to the fact that any nucleases allowed to persist in the cytosol would have access to host genomic DNA, the hydrolysis of which would be catastrophic for the cell. DNAse agar plate data (Fig. 1b) were consistent with the DSMFab strain nuclease being more active than the nucleases of the DSAFab and OSAFab strains. Relative to the other strains in this study, the greater activity of the ^DsbA^SMnuc in the DSMFab strain may exert a greater burden on the host cells with respect the cell maintenance requirement to ensure efficient translocation of the nuclease safely into the periplasmic space. Although this cell maintenance process is energetically affordable when the DSMFab strain is cultivated in complex media, the demands made by cultivation in defined media mean the cells must compromise cell growth in order to preserve cell maintenance, hence the observed growth retardation.

Li et al. (2014) showed that *E. coli* cultivation in complex versus defined media could have major global impacts on cell metabolism. Component molecules such as amino acids are already present in complex medium such as LB, so cells can simply utilise them directly for protein synthesis. By contrast, defined media such as the one used in this study, and the Defined Non-inducing Broth (DNB) used by Li et al. (2014), contain no amino acids so cells must devote energy to activities such as synthesis of component molecules such as amino acids in addition to polymerisation and folding of macromolecules such as proteins.

Given the observations of Li et al. (2014), and of Dragosits et al. (2012) discussed above, we suggest the different environments complex and defined media provide for *E. coli* cells could be sufficiently distinct that they would favour or inhibit growth in the same *E. coli* strain. Complex media enables cells to direct energy expenditure on protein production and folding to preserve cell maintenance. Defined media necessitates net diversion of energy away from cell maintenance toward biosynthesis of components such as amino acids.

The periplasm is the principle subcellular structure within didermal bacteria that defines their surface lipidome (Zückert 2014), glycome (Wang et al. 2016), secretome, nutrient uptake (Schalk and Guillon 2013; Sparacino-Watkins et al. 2014) and energy capture (Ishmukhametov et al. 2017). Dynamic control and remodelling of these processes can, therefore, be directly impacted by rates of protein translocation into and across the inner and outer membranes that define the periplasmic space. Directing recombinant proteins to the periplasm is likely to impact the performance of at least a subset of the multiple functions performed by the periplasm. This is frequently found to be the case for industrial strains of *E. coli* engineered to express recombinant periplasmic proteins. For such strains the outer membrane is prone to leak the majority of periplasmic contents to the external milieu during high cell density cultivation (Backlund et al. 2008).

Moving from OmpA to DsbA for the Staphylococcal nuclease rescued periplasmic Fab’ retention levels back to those of the original parental strain in which Fab’ fragment is expressed alone, with no co-expressed nuclease. We suggest that, in the OSAFab strain, routing three recombinant proteins using the OmpA signal (^*OmpA*^ *LC*, ^*OmpA*^ *HC* and ^*OmpA*^ *nucB*) compromised the performance of the SEC trafficking route, a downstream consequence of which was an increased propensity for outward leakage of periplasmic contents. Re-routing one of those three recombinant proteins away from the SEC pathway to the SRP pathway (^DsbA^nucB) was able to rescue the performance of the SEC pathway while at the same time preserving the performance of the SRP pathway. Further mechanistic studies will be needed to confirm this hypothesis.

We have shown that, when co-expressing a nuclease and a Fab’ fragment, the periplasm translocation route chosen for nuclease transport can be a significant factor in the performance of the strains so this should be considered an important focus of future mechanistic studies. These observations suggest large-scale screening of different nucleases, or variants of a given nuclease, is likely to identify further improvements in strain performance. Future biological investigation will identity which cellular limits, such as metabolic capacity or the carrying capacity of the periplasmic space, result in periplasmic content leakage to the external milieu when exceeded.
